# Accuracy of Transverse Cerebellar Diameter in Estimating Gestational Age in the Second and Third Trimester: A Prospective Study in Saudi Arabia

**DOI:** 10.3390/diagnostics15091130

**Published:** 2025-04-29

**Authors:** Awadia Gareeballah, Sultan Abdulwadoud Alshoabi, Ashwaq Mohammed Alharbi, Mashael Hisham Alali, Wed Mubarak Alraddadi, Fadwa Mohammed Al-Ahmadi, Reem Mustafa Dwaidy, Rahaf Alamri, Wessal Abdulkarim Alkhoudair, Walaa Alsharif, Maisa Elzaki, Amirah Faisal Alsaedi, Moawia Gameraddin, Osama Mohammed Abdulaal, Mohammed Adam

**Affiliations:** 1 Department of Diagnostic Radiology, College of Applied Medical Sciences, Taibah University, Al-Madinah Al-Munawwarah 42353, Saudi Arabiaal.ishwag1@gmail.com (A.M.A.); wsheref@taibahu.edu.sa (W.A.); elzakimaisa@gmail.com (M.E.); gameraldinm@gmail.com (M.G.); oabudulaal@taibahu.edu.sa (O.M.A.); 2 King Salman Medical City-Maternity and Children’s Hospital, Al-Madina Al-Munawwarah 42319, Saudi Arabia; fadwaa@moh.gov.sa (F.M.A.-A.); mrs.rahaf.1415@hotmail.com (R.A.);; 3 Department of Radiological Sciences, College of Applied Medical Sciences, King Khalid University, Abha 61421, Saudi Arabia; madam@kku.edu.sa

**Keywords:** gestational age (GA), last menstrual period (LMP), ultrasonography fetal measurements, transverse cerebellar diameter (TCD), biparietal diameter (BPD), femur length (FL)

## Abstract

**Background:** Failure to accurately estimate gestational age remains an important dilemma for optimal evidence-based antenatal care. Currently, when the last menstrual period (LMP) is unknown, ultrasonography measurement is the best method for estimating gestational age (GA). This study aims to assess the feasibility and accuracy of ultrasonography measurement of the transverse cerebellar diameter (TCD) to deduce fetal GA after 13 weeks of gestation. **Methods:** A prospective study was conducted on 384 normal singleton pregnancies. Demographic information and biometric measurements, including TCD, were collected using a data sheet. The data were then analyzed using SPSS version 27, DATAtab, and the R program. **Results:** The study found a strong significant association between GA based on TCD and the LMP, GA based on femur length (FL), GA based on biparietal diameter (BPD), GA based on abdominal circumference (AC), and GA based on the average gestational age (AVG) (r = 0.976, 0.970, 0.966, 0.968, and 0.984, respectively, *p* < 0.001). Furthermore, there was perfect agreement between GA estimated using TCD and GA based on LMP, with a mean difference of 0.41 weeks and upper and lower limits of agreement of −1.43 to 2.26 weeks. **Conclusions:** Ultrasonography measurements of the TCD accurately predict gestational age with excellent concordance with GA based on the LMP, FL, AC, and BPD. TCD can be used as a reliable estimator of GA in the second and third trimesters of pregnancy with the benefit of its brain-sparing effect in fetuses of fetal intrauterine growth restriction pregnancies. Combining TCD with FL, BPD, and AC provides the most accurate method of GA prediction.

## 1. Introduction

Precision in the assessment of gestational age (GA) is a crucial factor in managing antenatal care and calculating the expected date of delivery (EDD) appropriately [1]. Ultrasonography is considered the gold standard imaging method in antenatal care, with crown-rump length (CRL) measurements considered the most reliable in the first trimester. Biparietal diameter (BPD), femur length (FL), and abdominal circumference (AC) are the most used fetal biometric parameters in the second and third trimesters [2]. Classical fetal biometric parameters, such as mean gestational sac diameter, CRL, BPD, head circumference (HC), FL, and AC, are useful as the pregnancy progresses [3].

Unfortunately, all the fetal parameters mentioned are dependent on normal fetal growth. BPD, HC, FL, and AC are adversely affected in fetal intrauterine growth restriction (IUGR) [4,5], which affects up to 15% of pregnancies [5]. Furthermore, BPD is not reliable in fetuses with congenital head anomalies [6], and FL may be shorter and inaccurate in fetuses with genetic or developmental abnormalities [7].

The fetal cerebellum appears on ultrasonography as a butterfly structure with two symmetrically curved hemispheres conjoined by a hyperechoic cerebellar vermis with no change in appearance from gestational week 13 to week 39 [8]. Owing to the brain-sparing effect, the cerebellum is not affected in fetuses with IUGR [9]. Hence, ultrasonographic measurement of the fetal transverse cerebellar diameter (TCD) can offer a reliable estimator of GA in the second and third trimesters of pregnancy [10].

Many women do not have access to early antenatal care and attend hospitals in the second or third trimesters without any record of their LMP. This creates a dilemma of how to estimate the correct GA in these women to plan proper management of their pregnancy. The present study aims to assess the feasibility and accuracy of ultrasonography measurements of TCD to estimate fetal GA after 13 weeks of gestation. To the best of our knowledge, this is the first study to cover this topic in Saudi Arabia.

## 2. Materials and Methods

A prospective study was conducted on 384 normal singleton pregnancies admitted to the Maternity and Children Hospital in Al-Madina Al-Munawwarah, Kingdom of Saudi Arabia (KSA), for routine obstetric scanning during the study period of three months, from February to April 2024. The data were collected randomly using probability sampling techniques. The sample size was calculated based on an infinite population formula proposed by Almeda et al. [11] for quantitative research and resulted in 384 participants.

*n* = (Z^2^ σ^2^)/E^2^

where *n* is the sample size, Z is the Z-value (number of standard deviations from the mean corresponding to the desired confidence level, which is 1.96 for 95% confidence interval), σ is the population standard deviation, which is considered 0.5 (based on previous research), and E is the margin of error (the maximum acceptable difference between the sample mean and the population mean = 0.05) [11].

The study included all pregnant women with normal singleton pregnancies in their second and third trimesters who were sure of the date of the onset of LMP and who agreed to participate. Exclusion criteria included women with multiple pregnancies and fetal congenital anomalies, such as IUGR, women with irregular menstrual cycles, women who were unsure of the dates of their LMP and women with existing medical conditions or pregnancy related disorders. Data were collected using a specially designed data collection sheet. The sheet included the pregnant woman’s demographic data, measurement of GA using LMP, and ultrasonographic biometric measurements of AC, FL, BPD, and TCD.

### 2.1. Ethical Approval

This study was approved by the corresponding ethics committees of the College of Applied Medical Sciences at Taibah University and of King Salman bin Abdulaziz Medical City (No. IRB24-006). Verbal informed consent was obtained from each study participant.

### 2.2. Ultrasonography

Ultrasound examinations and TCD measurements were conducted by two skilled sonographers specializing in obstetric ultrasound, each with five years of experience (R.M.D, R.A). The accuracy of the measurements was confirmed by an experienced consultant in obstetrics (fetomaternal specialist) with six years of experience (F.M.A). A 3.5 MHz curvilinear transducer was used for obstetric examinations. The scan was conducted using two different ultrasound scanners—Voluson E10 and Voluson E6—manufactured by GE Healthcare in Austria, which employed the C1-5 curvilinear probe for the obstetric examination. These scanners provided various imaging modes, such as grayscale, Doppler, 3D, and 4D imaging, as well as elastography. The Voluson E10 was produced in 2017, while the Voluson E6 was produced in 2010.

Each pregnant female underwent scanning using one of scanner and no cross-scanning was conducted. We ensured that each scanner was calibrated according to standard protocols before data collection initiation, and both sonographers who conducted the ultrasound exam were trained using the same protocol to ensure consistency in measurements across them before starting the data collection (by a certified consultant fetomaternal specialist: F.M.A). Furthermore, in both rooms, the accuracy, consistency and correctness of the scanning protocol and measurements were confirmed by the same consultant fetomaternal specialist for all cases prior to interpretation of the results.

### 2.3. TCD Measures

**Figure 1:**
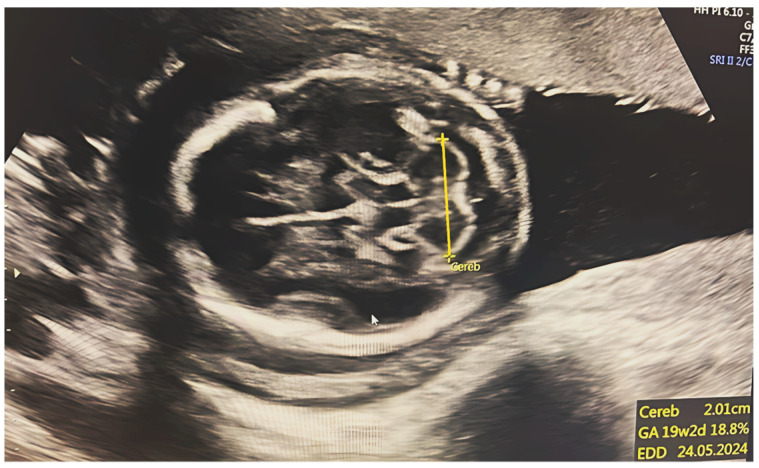
Selected ultrasonography image of transverse cerebellar dimension measurement to estimate fetal gestational age, which was found to be 19 weeks ± 2 days.

Initially, a comprehensive scan of the second and third trimesters was conducted, followed by a visualization of TCD. A suboccipitobregmatic view was used to measure TCD. This view allows a clear visualization of the anterior horns of the lateral ventricles, the cavum at the front of the head, and the cerebellum at the back, facilitating accurate measurement of the TCD. During TCD measurement, a 90° angle is formed with the long axis of the cerebellum extending across its widest point. The TCD measurement represents the distance between the outer boundaries of the two cerebellar hemispheres. Measurements are recorded in centimeters, and the device automatically calculates the gestational age of the cerebellum in weeks (Figure 1).

**Figure 2:**
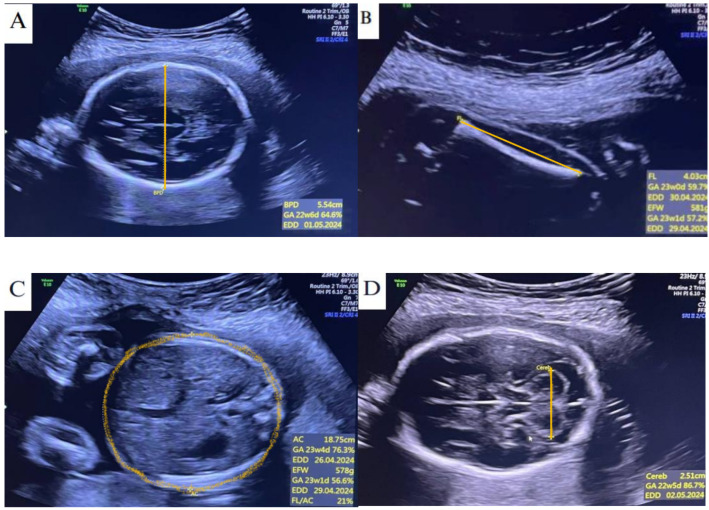
Selected ultrasonography image measurements of fetal gestational age (GA): (**A**) measurement of GA based on biparietal diameter (GA was 22 weeks ± 6 days); (**B**) GA based on femur length (23 weeks 0 day); (**C**) GA based on abdominal circumference (23 weeks ± 4 days); (**D**) GA based on transverse cerebellar diameter (22 weeks ± 5 days, respectively).

Ultrasonography biometric measurements of fetal GA using AC, FL, BPD, and TCD were performed on the same pregnant woman and the results were nearly equal (Figure 2).

### 2.4. Statistical Analysis

Collected data were analyzed using the Statistical Package for Social Sciences (SPSS) version 27 (IBM, Armonk, NY, USA), DATAtab Online Statistical Calculator 2024 (DATAtab e.U. Graz, Austria). Available online: https://datatab.net (accessed on 15 April 2024). Means and standard deviations were used to demonstrate continuous variables, frequency and percentage of categorical variables, and correlations and linear regression were applied to assess the relationship between TCD, and GA. R statistical software (Version 1.4.1103, R Foundation for Statistical Computing, Vienna, Austria) was used to obtain the percentile chart for TCD at differing GAs.

## 3. Results

The study included 384 participants aged 18–46 years (mean 31.74 ± 6.07). A total of 226 (68.2%) were in the second trimester of pregnancy (Table 1).

**Figure 3:**
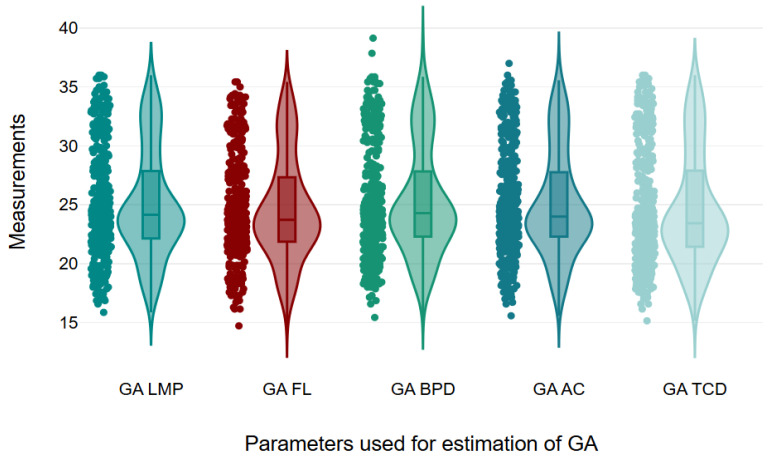
Violin plot showing assessment of GA using various parameters. GA = gestational age, LMP = last menstrual period, FL = femur length, BPD = biparietal diameter, AC = abdominal circumference, TCD = transcerebellar diameter.

In this study, the mean measurements for GA determined by LMP, FL, BPD, AC, average (AVG), and TCD were 25.05, 24.65, 25.26, 25.09, 24.98, and 24.64 weeks, respectively. In addition, the mean values for FL, BPD, AC, and TCD were 43.95 mm, 61.48 mm, 204.11 mm, and 28.15 mm, respectively. In the second trimester, the average GAs by LMP, FL, BPD, AC, AVG, and TCD were 22.40, 22.19, 22.60, 22.52, 22.34, and 21.9 weeks, respectively, with a mean TCD of 23.82 mm, while in the third trimester, the average GAs reported by LMP, FL, BPD, AC, AVG, and TCD were 30.74, 29.93, 30.98, 30.61, 30.66, and 30.51 weeks, respectively, with a mean TCD of 37.43 mm, and the mean TCD/AC ratio was 13.76 ± 0.90 (Table 2, Figure 3). 

**Figure 4:**
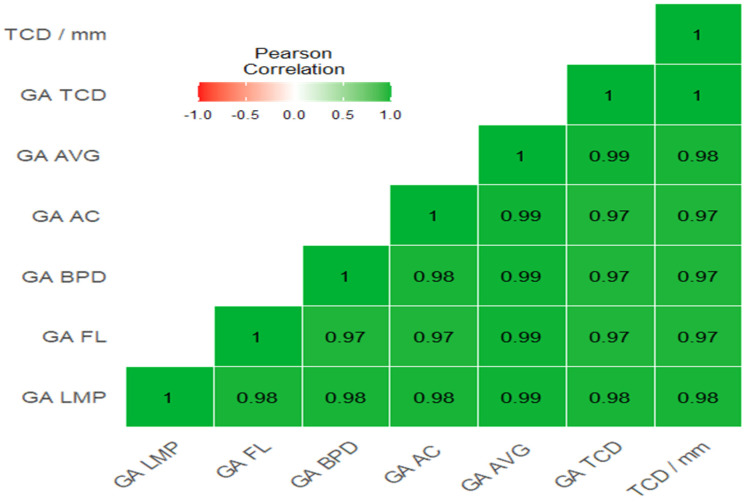
Pearson’s r heatmap for correlations between the study variables. GA = gestational age, AVG = average gestational age, LMP = last menstrual period, FL = femur length, BPD = biparietal diameter, AC = abdominal circumference, TCD = Transcerebellar diameter.

The study verified that GAs based on LMP, FL, BPD, AC, or average gestational age (AVG) from all parameters yielded a strong significant correlation with TCD mm (r = 0.976, 0.970, 0.966, 0.968, and 0.984, respectively, *p* < 0.001). A stronger correlation was also noted between the estimated GA based on TCD and LMP in relation to GA using BPD and LMP (r = 0.980 and 0.976, respectively, *p* < 0.001), and a significant but weak correlation was noted between the TCD/AC ratio and GA using varying parameters (*p* < 0.01) (Table 3, Figure 4).

Table 4 indicates that GA based on TCD demonstrates a higher correlation in the second trimester than in the third. In the second trimester, it was found that LMP, FL, BPD, AC, AVG, and TCD GAs yielded a strong significant correlation with TCD mm (r = 0.940, 0.929, 0.930, 0.925, 0.965, and 0.997, respectively), *p* < 0.001. In the third trimester, the LMP, FL, BPD, AC, AVG, and TCD GAs yielded a strong significant correlation with TCD mm (r = 0.919, 0.905, 0.856, 0.883, 0.942, and 0.996, respectively), *p* < 0.001.

**Figure 5:**
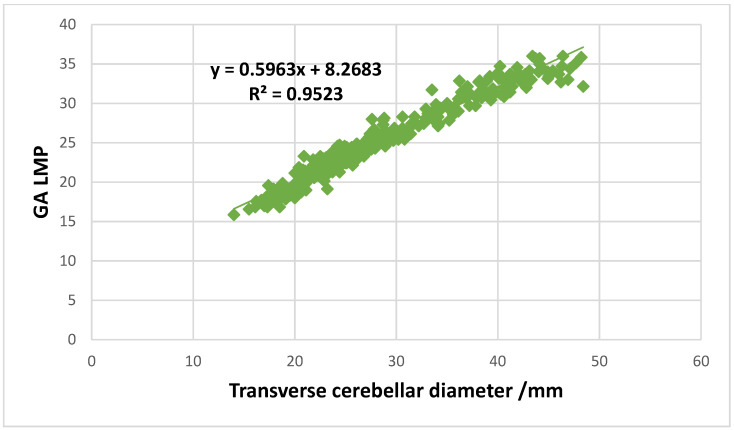
Scatter plot showing a strong linear association between transverse cerebellar diameter/mm and gestational age based on the date of the last menstrual period.

In the linear regression analysis, a strong positive linear association was observed between the TCD measurement/mm and GA based on LMP. The regression equations and corresponding r-squared values for each association are as follows: GA LMP = 0.5963, TCD/mm + 8.2683 (R^2^ = 0.9523) (Figure 5).

Bland and Altman’s analysis found that the mean difference (bias) between GA based on LMP and GA based on TCD was 0.41 weeks, with 4.43% of measurements lying outside the limit of agreement (17/384 × 100), and the margin of error was −1.43 to 2.26 (the GA estimated by LMP was 1.43 weeks below or 2.26 weeks higher than the GA estimated based on TCD). The mean difference between GA based on LMP and on FL was 0.40 weeks, with 17 points (4.43%) lying outside the limit of agreement, and the margin of error was −1.38 to 2.18 (the GA calculated by LMP was 1.38 weeks below or 2.18 weeks higher than the GA FL). The mean difference between GA based on LMP and GA based on BPD was −0.21 weeks, with 3.91% of cases lying outside the limit of agreement (15/384 × 100); the margin of error was −2.22 to 1.80 (the GA calculated by LMP was 2.22 weeks less or 1.80 weeks higher than the GA estimated by BPD).

**Figure 6:**
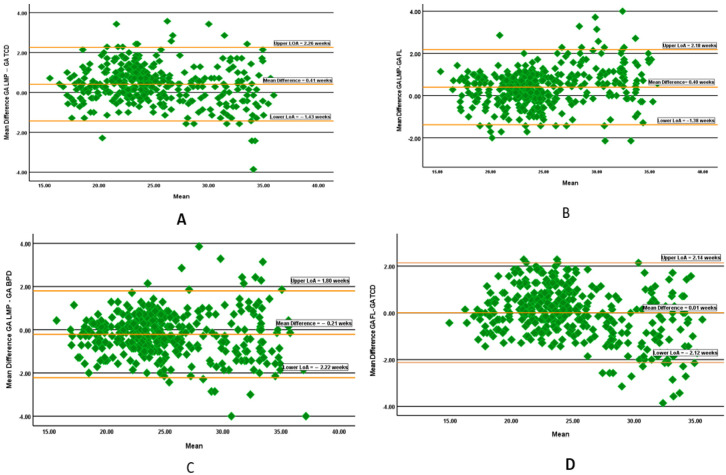
Bland and Altman’s analysis of the agreement between gestational age (GA) (**A**) based on the last menstrual period (LMP) and on TCD, (**B**) based on LMP and GA using femur length (FL), (**C**) based on LMP and GA using biparietal diameter (BPD), and (**D**) based on femur length (FL) and TCD.

On the other hand, the mean difference between GA based on FL and GA based on TCD was only 0.01, with an upper and lower limit of agreement of −2.12 to 2.14 (the GA calculated by FL was 2.12 weeks below or 2.14 weeks higher than the GA estimated by TCD); only 3.64% of measurements lie outside the limit of agreement (14/384 × 100) (Figure 6).

A linear regression model shows that combination of TCD/mm with other parameters (BPD, AC, and FL) demonstrate greater accuracy in estimation of GA (98%) (the regression model was as follows: GALMP = 6.31 + 0.12·FL/mm + 0.05·BPD/mm + 0.03·AC/mm + 0.19·TCD/mm; F = 4338.57, *p* = <0.001, R^2^ = 0.98), than using combination of routine parameters alone (BPD, FL, and AC) (97%); GA LMP = 5.77 + 0.16·FL/mm + 0.07·BPD/mm + 0.04·AC/mm; F = 4641.5, R^2^ = 0.97. Furthermore, for estimation of GA LMP using single parameters AC and FL produce the same accuracy (96%; R^2^ = 0.96), followed by TCD (95.2%; R^2^ = 0.952) then BPD (95%; R^2^ = 0.95) Table 5.

**Figure 7:**
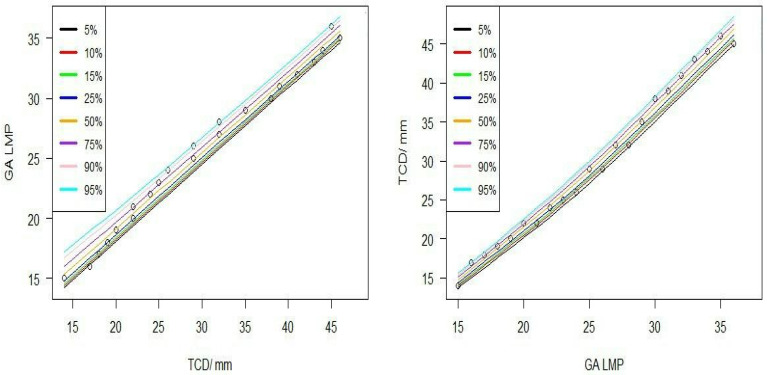
Percentile charts for the means of transverse cerebellar diameter at various gestational ages based on the last menstrual period (from 15 weeks and 6 days [15 + 6] to 36 weeks of pregnancy).

The nomogram in Figure 7 shows that TCD is an accurate parameter for GA estimation. The chart shows the 5th, 10th, 15th, 25th, 50th, 75th, 90th, and 95th percentiles for TCD evaluated for a range of GAs based on LMP.

## 4. Discussion

This study aimed to assess the accuracy of ultrasonography measurements of TCD to estimate fetal GA after 13 weeks of gestation. The findings of our study determined the strongest Pearson’s coefficient correlation (r = 0.980, *p* < 0.001) by comparing the estimated GA based on TCD and on LMP. This aligns with a previous study that reported that TCD was the best parameter to correlate with GA by LMP [12]. Negele’s rule relies on biologically questionable assumptions, including that LMP is the appropriate time zero of pregnancy, and ovulation occurs on day 14 of a 28-day menstrual cycle. However, the date of the onset of LMP may be poorly recalled, the menstrual cycle may be irregular, or the time of ovulation may vary, even in women with regular menstrual cycles [13]. Regarding methods for estimating GA and the due date, the American College of Obstetricians and Gynecologists, the American Institute of Ultrasound in Medicine, and the Society of Maternal—Fetal Medicine recommend the following: as soon as data from the LMP or the first accurate ultrasonography, or both, are available, the GA should be documented in the patient’s medical record, and subsequent changes to the EDD (Expected Date of Delivery) should be reserved for rare circumstances discussed with the patient and documented prominently in her medical record [14].

The findings of our study proved that GA based on BPD yielded a strong significant correlation with GA based on TCD (r = 0.966, *p* < 0.001). This result is compatible with a previous study that reported a strong correlation between GA based on BPD and TCD, with r = 0.951 in the second and third trimesters of pregnancy [15]. Johnsen et al. reported that in the second trimester, both HC and BPD predicted EDD with a mean accuracy of one day, and HC was significantly more accurate than BPD [16]. This means that both HC and BPD are accurate measurements for estimating GA in the second trimester. Our results proved that TCD is another parameter to accurately estimate GA in both the second and third trimesters. Another study reported that GA based on TCD was a more reliable method than BPD for estimations in the third trimester [17].

The findings of this study affirm that GA based on FL yields a strong significant correlation with GA based on TCD (r = 0.970, *p* < 0.001). This finding is strongly compatible with a previous study that reported a strong correlation between GA based on FL and TCD with r = 0.981 in the second and third trimesters of pregnancy [15]. In the second trimester, combining FL and HC (Head Circumference) can be used to estimate GA with reasonable accuracy [18]. In the third trimester, combining FL and HC is more accurate for estimating GA than the FL measurement alone [19]. The World Health Organization (WHO) Alliance for Maternal and Newborn Health Improvement’s Late Pregnancy Dating Study Group reported that incorporating TCD and the use of new formulas in late pregnancy ultrasonography can improve the accuracy of GA dating in both appropriate-for-GA and small-for-GA infants [20]. TCD can be used as a reliable parameter for the evaluation of GA when the LMP is not known [21].

In this study, it was found that the mean differences between GA estimated by LMP and GA estimated by TCD was 0.41 weeks, with an upper and lower limit of agreement of −1.43 to 2.26, and the mean differences between GA estimated by FL and GA TCD was 0.01 weeks, with an upper and lower limit of agreement of −2.12 to 2.14 weeks. Mahmood et al. mention that GA estimated by TCD differed only −7 to 6 days from actual gestational age across various trimesters of pregnancy [22].

The study demonstrated that TCD can be used as predictor for GA, with strong positive relationship noted between TCD and GA LMP (GA LMP = 0.5963 × TCD/mm + 8.2683, R^2^ = 0.9523). Prasad et al., consistent to this study, mentioned that TCD can be used as predictor for GA estimation, with a strong relationship between GA and TCD (GA = 2.21 + 0.95 × (TCD) − 0.00404 × (TCD)^2^, R^2^ = 0.989; *p* < 0.001) which was similar to GA assessed by BPD, HC, and AC [23].

The growth of the cerebellum in humans is less affected by IUGR (Intrauterine growth restriction), so TCD measurements are a reliable predictor of gestational age. IUGR fetuses typically have lower levels of hepatic glycogen and subcutaneous fat, leading to a decrease in abdominal circumference (AC). Therefore, AC measurements are a sensitive indicator of fetal IUGR. The TCD/AC ratio is the least affected biometric parameter and may be a valuable tool for detecting asymmetric IUGR at any stage of pregnancy [24]. The study found that the mean TCD/AC ratio’s percentage was 13.76 ± 0.90, with a cut-off value of 15.56% can be used for diagnosing IUGR. This finding aligns with Kadiyala et al., Tongsong et al., and Campbell et al., who reported cut-off values of TCD/AC ratio of 15.49%, 15.4%, and 15.9%, respectively [25,26,27].

Ultimately, ultrasonography offers a real-time, non-invasive, widely available, radiation-free, and easy-to-use imaging modality [28] that can be used in measuring fetal biometric parameters for fetal age estimation based on other body organs with good accuracy [29], such as fetal kidney length [3], fetal humeral length, and placental thickness, as a reliable method in singleton pregnancies [30]. Finally, machine learning based on ultrasonography images is a promising method for accurate estimation of GA [31]. Artificial intelligence (AI) is emerging as a powerful tool for enhancing various domains of maternal healthcare [32].

The generalizability of these results is limited by the fact that participants in this study were from a single center and that the sample size was relatively small. Future multicenter studies with large sample sizes are recommended. Furthermore, a case control study to compare the TCD/AC ratios in a normal and IUGR fetus.

## 5. Conclusions

Ultrasonography measurements of the TCD provide an accurate prediction of gestational age with excellent concordance with GA based on the LMP, FL, and BPD. TCD can be used as a reliable estimator of GA in the second and third trimesters of pregnancy, with the benefit of its brain-sparing effect in fetuses with IUGR. Combining TCD with FL, BPD, and AC provides the most accurate method of GA prediction.

## Figures and Tables

**Table 1:** Frequency distribution of age groups/years and trimester of the pregnancy.

Demographic Characteristics		Frequency	Percent
Age groups/years	18–32	213	55.5
	33–46	171	44.5
	Mean ± Std. = 31.74 ± 6.07		
Trimester of the pregnancy	Second trimester	262	68.2
	Third trimester	122	31.8
Total		384	100.0

**Table 2:** Mean measurements and 95% CI of mean parameters used for estimating GA and GA using TCD in two pregnancy trimesters.

Study Variables	Trimester	Frequency	Mean ± Std.	95% Confidence Interval for Mean
**BPD/mm**	Second	262	54.27 ± 7.2	53.39–55.15
	Third	122	76.98 ± 7.45	75.65–78.32
	Total	384	61.49 ± 12.84	60.19–62.78
**AC/mm**	Second	262	175.97 ± 26.29	172.75–179.18
	Third	122	264.56 ± 30.07	259.16–269.96
	Total	384	204.11 ± 49.62	199.11–209.12
**FL/mm**	Second	262	37.92 ± 6.45	37.13–38.71
	Third	122	56.9 ± 6.46	55.74–58.06
	Total	384	43.95 ± 10.95	42.85–45.06
**TCD/mm**	Second	262	23.82 ± 3.26	23.43–24.22
	Third	122	37.43 ± 5.49	36.44–38.42
	Total	384	28.15 ± 7.55	27.39–28.91
**GA LMP**	Second	262	22.4 ± 2.32	22.12–22.69
	Third	122	30.74 ± 2.82	30.23–31.25
	Total	384	25.05 ± 4.61	24.59–25.52
**GA FL**	Second	262	22.19 ± 2.36	21.9–22.48
	Third	122	29.93 ± 2.85	29.42–30.44
	Total	384	24.65 ± 4.4	24.2–25.09
**GA BPD**	Second	262	22.6 ± 2.32	22.32–22.89
	Third	122	30.98 ± 3.02	30.43–31.52
	Total	384	25.26 ± 4.67	24.79–25.74
**GA AC**	Second	262	22.52 ± 2.3	22.24–22.8
	Third	122	30.61 ± 2.84	30.1–31.12
	Total	384	25.09 ± 4.51	24.64–25.55
**GA AVG**	Second	262	22.34 ± 2.27	22.06–22.62
	Third	122	30.66 ± 2.88	30.15–31.18
	Total	384	24.98 ± 4.6	24.52–25.45
**GA/TCD**	Second	262	21.9 ± 2.25	21.63–22.18
	Third	122	30.51 ± 3.1	29.95–31.07
	Total	384	24.64 ± 4.75	24.16–25.12
**TCD/AC Ratio**	Second	262	13.60 ± 0.81	13.50–13.69
	Third	122	14.12 ± 0.97	13.94–14.29
	Total	384	13.76 ± 0.90	13.67–13.85

GA: gestational age, LMP: last menstrual period, FL: femur length, BPD: biparietal diameter, AC: abdominal circumference, AVG: average (The average of GA calculated by US), TCD: transverse cerebellar diameter, TCD/AC ratio is transverse cerebellar diameter/abdominal circumference × 100%, Second: second trimester, Third: Third trimester.

**Table 3:** Correlation of GA LMP, TCD/mm, GA TCD, and TCD/AC ratio with GA based on LMP, FL, BPD, AC, and AVG/weeks.

Parameter of GA Estimation	Gestational Age Parameter/Weeks					*p*-Value
	LMP	FL	BPD	AC	AVG	
	Pearson (r)					
**GA LMP**	1	0.981 **	0.976 **	0.981 **	0.989 **	<0.001
**GA TCD**	0.980 **	0.975 **	0.971 **	0.973 **	0.988 **	
**TCD/mm**	0.976 **	0.970 **	0.966 **	0.968 **	0.984 **	
**TCD/AC Ratio**	0.233 **	0.225 **	0.208 **	0.137 **	0.344 **	<0.01

** Correlation is significant at the 0.01 level (2-tailed). r: correlation coefficient, GA: gestational age, TCD: transverse cerebellar diameter, LMP: last menstrual period, FL: femur length, BPD: biparietal diameter, AC: abdominal circumference, mm: millimeter, AVG: average gestational age, GA TCD: gestational age by transverse cerebellar diameter.

**Table 4:** Correlation of TCD/mm and the GAs of LMP, FL, BPD, AC, AVG, and TCD in the second and third trimesters.

Trimester of the Pregnancy		Gestational Age per Different Parameters/Weeks						*p*-Value
		LMP	FL	BPD	AC	AVG	TCD	
		Pearson (r)						
**Second**	**GA LMP**	1	0.948 **	0.942 **	0.942 **	0.966 **	0.941 **	<0.001
	**TCD/mm**	0.940 **	0.929 **	0.930 **	0.925 **	0.965 **	0.997 **	
	**GA TCD**	0.941 **	0.932 **	0.933 **	0.927 **	0.966 **	1	
**Third**	**GA LMP**	1	0.928 **	0.886 **	0.924 **	0.954 **	0.923 **	<0.001
	**TCD/mm**	0.919 **	0.905 **	0.856 **	0.883 **	0.942 **	0.996 **	
	**GA TCD**	0.923 **	0.910 **	0.861 **	0.890 **	0.948 **	1	

** Correlation is significant at the 0.01 level (2-tailed). r: correlation coefficient, GA: gestational age, TCD: transverse cerebellar diameter, LMP: last menstrual period, FL: femur length, BPD: biparietal diameter, AC: abdominal circumference, mm: millimeter, AVG: average gestational age.

**Table 5:** Regression model for assessment of accuracy and prediction of GA using TCD, FL, BPD, and AC in comparison to prediction based on combination of routine parameters and using TCD combined with routine parameters.

Method of Estimation		Unstandardized Coefficients	Standardized Coefficients	t	*p*-Value	95% CI for B	F	R^2^
	**Model**	B	Beta					
**BPD, FL, AC, TCD/mm**	**Constant**	6.31		29.83	<0.001	5.89–6.73	4338.5	0.98
	**FL/mm**	0.12	0.27	7.24	<0.001	0.08–0.15		
	**BPD/mm**	0.05	0.13	3.25	0.001	0.02–0.08		
	**AC/mm**	0.03	0.29	6.54	<0.001	0.02–0.03		
	**TCD/mm**	0.19	0.31	9.6	<0.001	0.15–0.23		
**GA LMP = 6.31 + 0.12·FL/mm + 0.05·BPD/mm + 0.03·AC/mm + 0.19·TCD/mm**								
**BPD, FL, AC/mm**	**(Constant)**	5.77		25.42	<0.001	5.32–6.21	4641.5	0.97
	**FL/mm**	0.16	0.38	9.48	<0.001	0.13–0.19		
	**BPD/mm**	0.07	0.19	4.11	<0.001	0.03–0.1		
	**AC/mm**	0.04	0.43	9.26	<0.001	0.03–0.05		
**GA LMP = 5.77 + 0.16·FL/mm + 0.07·BPD/mm + 0.04·AC/mm**								
**TCD/mm**	**(Constant)**	8.267		41.534	<0.001	7.87–8.65	7620.5	0.952
	**TCD/mm**	0.596	0.976	87.296	<0.001	0.58–0.61		
**GA LMP = 8.27 + 0.6·TCD/mm**								
**BPD/mm**	**Constant**	3.51		13.77	<0.001	3.01–4.02	7433.8	0.95
	**BPD/mm**	0.35	0.98	86.22	<0.001	0.34–0.36		
**GA LMP = 3.51 + 0.35·BPD/mm**								
**AC/mm**	**Constant**	6.44		32.95	<0.001	6.06–6.83	8473.3	0.96
	**AC/mm**	0.09	0.98	97.92	<0.001	0.09–0.09		
**GA LMP = 6.44 + 0.09·AC/mm**								
**FL/mm**	**Constant**	6.93		34.17	<0.001	6.53–7.33	9588.1	0.96
	**FL/mm**	0.41	0.98	92.05	<0.001	0.40–0.42		
**GA LMP = 6.93 + 0.41·FL/mm**								

## Data Availability

Data are available from the corresponding author upon reasonable request.

## Abbreviations

The following abbreviations are used in this manuscript:

AC	Abdominal Circumference
AVG	Average
AI	Artificial Intelligence
BPD	Biparietal Diameter
EDD	Expected Date of Delivery
FL	Femur Length
GA	Gestational Age
HC	Head Circumference
IUGR	Intrauterine Growth Restriction
LMP	Last Menstrual Period
mm	millimeter
TCD	Transverse Cerebellar Diameter
WHO	World Health Organization
